# Superficial Acral Fibromyxoma of Ring Finger: A Rare Entity and Its Management

**DOI:** 10.1055/s-0046-1816054

**Published:** 2026-02-09

**Authors:** Vijay Kumar, Mukta Verma, Kartikeya Shukla, Sandhya Pandey, Bhasker Chowdhary, Vidush Kumar

**Affiliations:** 1 Department of Plastic and Reconstructive Surgery, King George's Medical University, Lucknow, Uttar Pradesh, India

**Keywords:** superficial acral fibromyxoma (SAFM), benign, finger swelling

## Abstract

**Background:**

Superficial acral fibromyxoma (SAFM) is a rare, benign fibroblastic neoplasm. Periungual and subungual regions of the acral sites are involved predominantly. These lesions are slow growing and have a high propensity for local recurrence.

**Materials and Methods:**

A 41-year-old female presented as painless, slowly enlarging mass in the periungual region in the ulnar aspect of the ring finger. Radiological evaluation was done.

**Results:**

The patient underwent wide local excision of the lesion with subsequent reconstruction of the surgical defect with homodigital flap and split-thickness skin graft over the residual raw area. Histopathological analysis confirmed the diagnosis. Postoperative recovery was uneventful, and no evidence of recurrence was observed at short-term follow-up.

**Conclusion:**

SAFM, though uncommon, should be considered in the differential diagnosis of acral soft tissue tumors. Complete surgical excision with adequate margins remains the mainstay of treatment to minimize recurrence. This case highlights the importance of clinicopathological correlation and long-term follow-up in managing such rare tumors.

## Introduction

Digital fibromyxoma, formally termed superficial acral fibromyxoma (SAFM), is a rare, nonmalignant tumor affecting the acral regions. It is mainly slow-growing, found in middle- aged adults and is a benign neoplasm. 1 Fetsch et al reported SAFM in 2001. 2 It often presents as solitary soft tissue swelling over the periungual or subungual regions of the digits on acral sites. 2 These lesions are prone to local recurrence.

## Case Review

A 41-years-old female presented in the outpatient department (OPD) with fleshy outgrowth of the right ring finger for 1 year. It began with small pea-sized swelling over the base of the right ring finger, progressively increasing in size and painless; swelling was initially ignored by the patient as it was not hampering her routine. Later on, patient sought medical advice from a nearby medical practitioner 4 months back and some surgical intervention was performed, since then there was a wound with persistent serosanguineous discharge. As per the patient, no tissue was sent for histopathological examination and no records were available for the same. The patient was otherwise well and was on regular dressings and she was referred to us for wound management. There was no history suggestive of trauma, diabetes mellitus type 2, or hypertension.

**Fig. 1:**
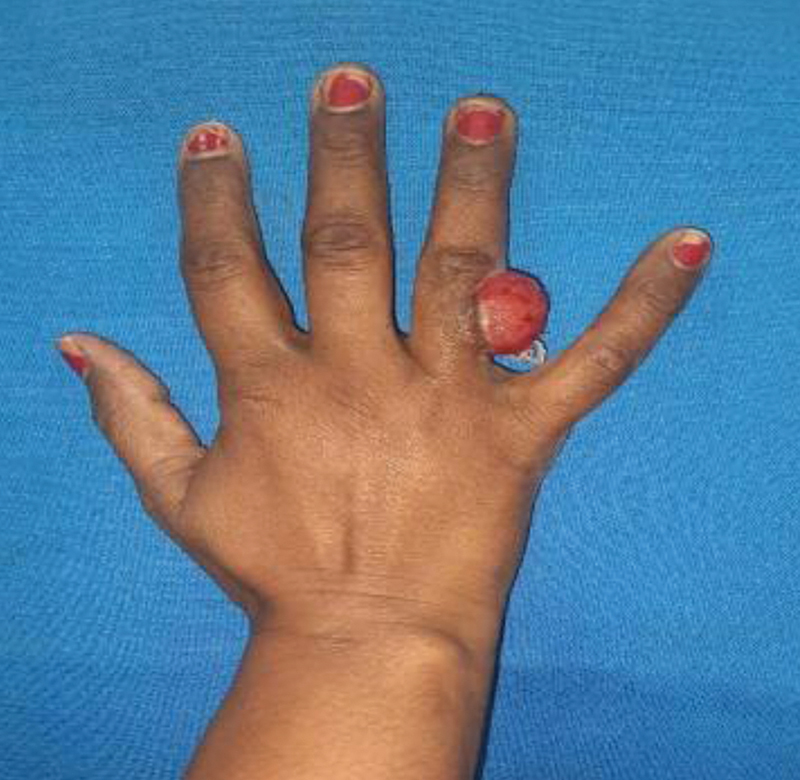
Ulceroproliferative swelling at the base of the right ring finger.

On examination, there was a 2 cm × 2 cm ulceroproliferative swelling over the proximal phalanx of the right ring finger. Swelling was well defined, painless, and firm in consistency ( Fig. 1 ).

No bony abnormality was detected on radiological examination (X-ray). Patient was planned for excisional biopsy and flap cover under brachial block.

## Surgical Management

**Fig. 2:**
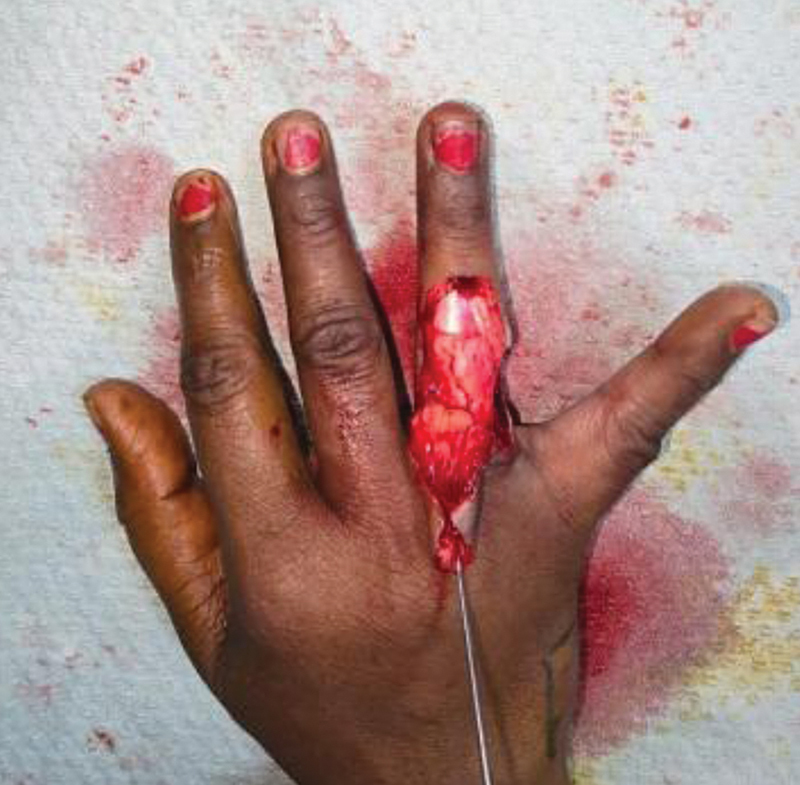
Intraoperative photograph showing: ( **A** ) The defect after excision of swelling over the ulnar aspect of the proximal phalanx of the right ring finger. ( **B** ) Elevated homodigital flap for defect coverage (elevated with skin hook as shown in the figure. ( **C** ) Split-thickness skin graft donor site marked with methylene blue dye over ulnar aspect of the hypothenar area of the right hand.

**Fig. 3:**
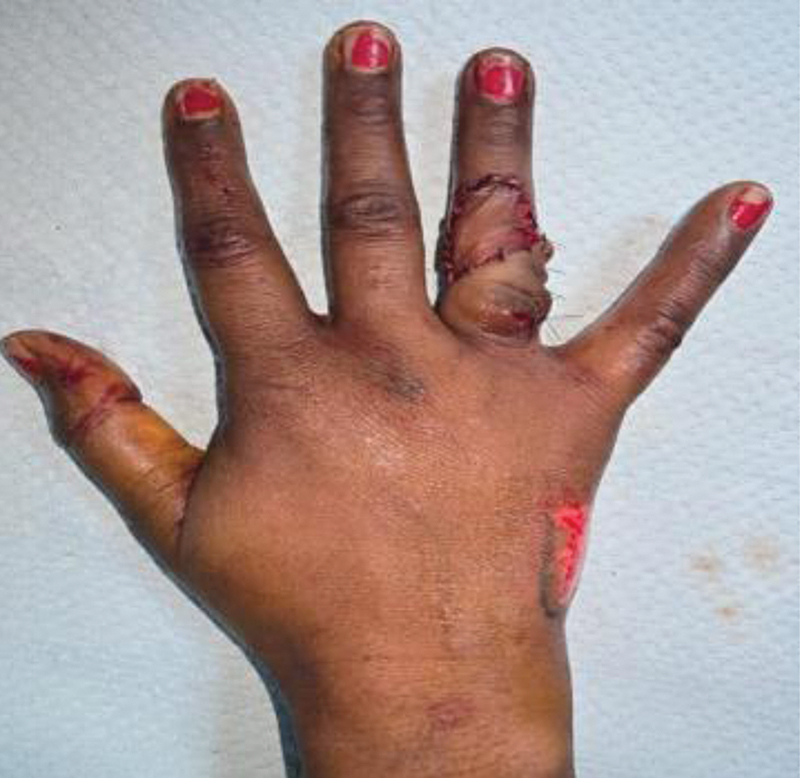
Intraoperative photograph showing the defect coverage with homodigital flap and secondary raw area coverage with split-thickness skin graft (STSG), which was taken from the ulnar aspect of the same hand.

Under right brachial block with tourniquet control and magnification, swelling was excised as wide excisional biopsy with adequate margins with one plane deeper. Resultant raw area was covered with homodigital flap and secondary raw area was covered with split-thickness skin graft taken from the hypothenar area of the same hand. The excised tissue was then sent for histopathological examination ( Figs. 2 and 3 ).

## Histopathological Details

Microscopic examination and immunohistochemistry (IHC) were performed.

**Fig. 4:**
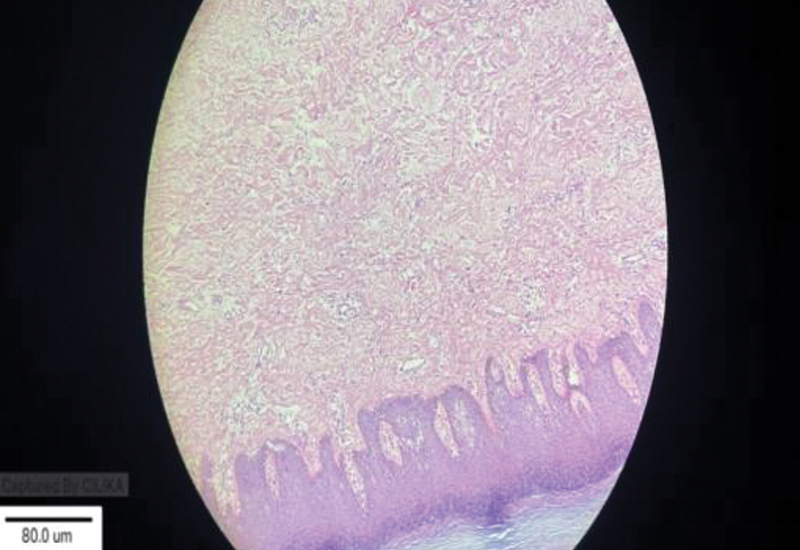
Microscopic image showing spindle- to stellate-shaped fibroblastic cells arranged in a loose storiform pattern with alternating myxoid and collagenous stroma.

Histological examination demonstrated a benign, ill-defined lesion within the dermis consisting of fibroblastic cells with spindle to stellate morphology. These were arranged in a loose storiform configuration amidst alternating myxoid and collagen-rich stroma. There was no evidence of nuclear or cytological atypia. There was prominent thin vasculature. Scattered mast cells were also noticed. Margin was cleared ( Fig. 4 ).

Tumor cells were found to be positive for CD34, CD99, and smooth muscle actin (SMA) and were negative for BCL2, so IHC evaluation pointed toward the diagnosis of SAFM.

**Fig. 5:**
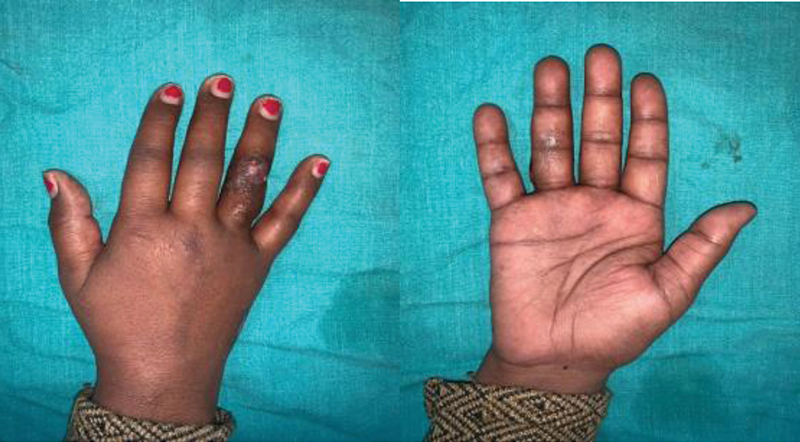
Postoperative photograph with dorsal and ventral profile of the right hand at 3 weeks showing satisfactory recovery with normal finger contour and healed split-thickness skin graft (STSG) donor site.

**Fig. 6:**
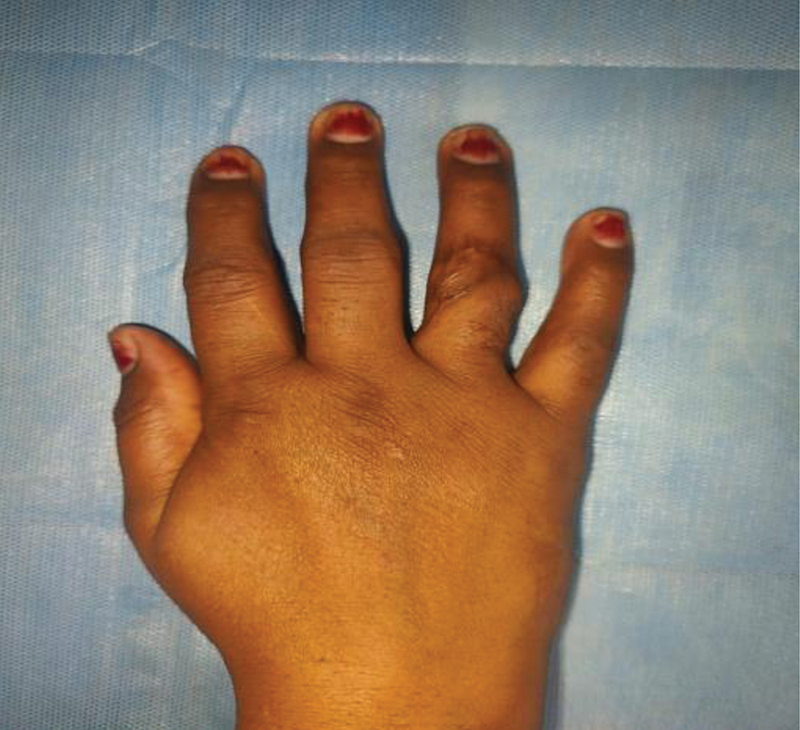
Postoperative photograph with dorsal profile of the right hand at 6 months' follow-up showing satisfactory recovery with minimal scarring and good functional outcome.

Postoperative recovery was satisfactory. Patient was discharged on the same day of surgery and was followed up in the OPD. Sutures were removed on postoperative day 10, following which she was advised for scar massage and follow-up at regular intervals for better recovery ( Figs. 5 and 6 ).

## Discussion and Review of Literature

The etiopathology of SAFM is not well established. Clinically, its main presentation is solitary nodular swelling with a higher predilection for the acral sites, mainly occurring in the periungual/subungual regions. 2 3 4 Few cases have been reported in other areas, such as the hand, thigh, leg, heel, and foot. Male-to-female ratio is 2:1, and is mainly found in male patients of middle age. SAFM is a slow-growing, benign mesenchymal tumor. 4 5 Still due to pressure effect, erosive or lytic lesion of the underlying bone may be seen on imaging in approximately 36% of cases. 6 7

Ultrasound examination, being a noninvasive radiological tool, can provide additional important information regarding tumor size, location, and vascularity. Recurrences can also be monitored by ultrasound. 8

Initially, plain X-ray is done if any bony abnormality or bony erosion is detected, then magnetic resonance imaging or computed tomography (CT) scan can be done. CT scan can reveal calcifications within soft tissues as well as erosive changes in the bone accurately. 9

Surgical excision with adequate margin is the treatment of choice. Incomplete resection leads to recurrence and delay in complete recovery. 5 10 11 Incomplete excisions are common over fingers and toes due to paucity of tissue after tumor excision for primary closure, so graft/flap cover should be done wherever required.

Multidisciplinary approach (surgeon/plastic and reconstructive surgeon, radiologist, pathologist, and physiotherapist) is needed for proper management.

Histological examination of the lesion is diagnostic. Oval to spindle cells with bland nuclear chromatin with mucoid/myxoid material background and groups/cohesive clusters of spindle cells with scattered thin-walled capillaries is diagnostic of SAFM. 2 10 Dermatoscopic appearance shows a nonpigmented tumor, appearing pink, homogeneous color, and longitudinal, soft-pink, and small, shiny, and white streaks. 12

Local recurrence is uncommon following complete excision.

*Differential diagnosis* may be myxoid neurofibroma (prominent vasculature is lacking, S100 and CD34 positive); superficial angiomyxoma (mostly affecting the head and neck region, SMA and CD34 is negative); acral fibrokeratoma (polypoid dome-shaped, dense collagenous stroma, mature spindle cells, and small vessels, vertical orientation of collagen fibers); dermatofibrosarcoma protuberans (mainly affecting trunk and extremities, CD34 is positive); fibroma of tendon sheath (well circumscribed, attached to tendon, SMA is positive, CD34 is negative); giant cell tumor of tendon sheath (attached to tendon sheath, spindle, epithelioid and giant cells in fibrous stroma, CD34 is negative and CD68 is positive); glomus tumor (solitary painful mass of digits, uniform round to polygonal cells arranged around vessels, SMA is positive, CD34 is negative). 2 11

## Conclusion

SAFM, a rare benign mesenchymal neoplasm, is best managed through wide local excision with histologically clear margins, accompanied by regular postoperative monitoring and is recommended to find out future recurrences early. Incomplete excision leads to mismanagement and delay in complete recovery. When dealing with soft tissue tumors on the hands or feet, SAFM should be kept in mind, especially if the tumor has recurred following surgery. This case also shows how important it is to involve multiple specialists in the diagnosis and treatment.

## References

[1] Altman R D Tenenbaum J Hypertrophic osteoarthropathy Philadelphia WB Saunders Company 1997 1514 1520

[2] Fetsch J F Laskin W B Miettinen M Superficial acral fibromyxoma: a clinicopathologic and immunohistochemical analysis of 37 cases of a distinctive soft tissue tumor with a predilection for the fingers and toes Hum Pathol 2001 32 07 704 714 11486169 10.1053/hupa.2001.25903

[3] Ashby-Richardson H Rogers G S Stadecker M J Superficial acral fibromyxoma: an overview Arch Pathol Lab Med 2011 135 08 1064 1066 21810002 10.5858/2009-0684-RSR1

[4] Sinha A Phukan J P Bandopadhyay G Sengupta S Saha R Superficial acral fibromyxoma: a rare tumor diagnosed by cytology Iran J Pathol 2013 8 59 63

[5] Hankinson A Holmes T Pierson J Superficial acral fibromyxoma (digital fibromyxoma): a novel treatment approach using Mohs micrographic surgery for a recurrence-prone digital tumor Dermatol Surg 2016 42 07 897 899 27191786 10.1097/DSS.0000000000000735

[6] Hashimoto K Nishimura S Oka N Tanaka H Kakinoki R Akagi M Aggressive superficial acral fibromyxoma of the great toe: a case report and mini-review of the literature Mol Clin Oncol 2018 9 03 310 314 30112176 10.3892/mco.2018.1669 PMC6090573

[7] Hollmann T J Bovée J VMG Fletcher C DM Digital fibromyxoma (superficial acral fibromyxoma): a detailed characterization of 124 cases Am J Surg Pathol 2012 36 06 789 798 22367301 10.1097/PAS.0b013e31824a0b83

[8] Baek H J Lee S J Cho K H Subungual tumors: clinicopathologic correlation with US and MR imaging findings Radiographics 2010 30 06 1621 1636 21071379 10.1148/rg.306105514

[9] Mundada P Becker M Lenoir V High resolution MRI of nail tumors and tumor-like conditions Eur J Radiol 2019 112 93 105 30777226 10.1016/j.ejrad.2019.01.004

[10] WHO Classification of Tumours Editorial Board Soft Tissue and Bone Tumours. WHO Classification of Tumours Series. 5th ed. Vol. 3 Lyon, France International Agency for Research on Cancer 2020

[11] Luzar B Calonje E Superficial acral fibromyxoma: clinicopathological study of 14 cases with emphasis on a cellular variant Histopathology 2009 54 03 375 377 19236516 10.1111/j.1365-2559.2008.03209.x

[12] Sialitti S Abdelmouttalib A Senouci K Meziane M Subungual acral fibromyxoma: new dermoscopic features Our Dermatol Online 2021 12 1 2

